# Mask images on Twitter increase during COVID-19 mandates, especially in Republican counties

**DOI:** 10.1038/s41598-022-23368-6

**Published:** 2022-12-09

**Authors:** Xiaofeng Lin, Georgia Kernell, Tim Groeling, Jungseock Joo, Jun Luo, Zachary C. Steinert-Threlkeld

**Affiliations:** 1 Department of Statistics, University of California - Los Angeles, Los Angeles, CA 90024 USA; 2 Department of Communication, University of California - Los Angeles, Los Angeles, CA 90024 USA; 3 Department of Political Science, University of California - Los Angeles, Los Angeles, CA 90024 USA; 4 Department of Public Policy, Luskin School of Public Affairs, University of California - Los Angeles, Los Angeles, CA 90024 USA

**Keywords:** Information technology, Public health, Human behaviour

## Abstract

Wearing masks reduces the spread of COVID-19, but compliance with mask mandates varies across individuals, time, and space. Accurate and continuous measures of mask wearing, as well as other health-related behaviors, are important for public health policies. This article presents a novel approach to estimate mask wearing using geotagged Twitter image data from March through September, 2020 in the United States. We validate our measure using public opinion survey data and extend the analysis to investigate county-level differences in mask wearing. We find a strong association between mask mandates and mask wearing—an average increase of 20%. Moreover, this association is greatest in Republican-leaning counties. The findings have important implications for understanding how governmental policies shape and monitor citizen responses to public health crises.

## Introduction

Widespread mask wearing greatly reduces COVID-19 transmission^1–6^. Accurate and continuous measures of mask use are therefore necessary for public health agencies to understand and predict outbreaks, identify susceptible populations, and formulate timely policy responses. Throughout the COVID-19 pandemic, health and government officials, as well as the general public, received real-time access to important information such as hospitalizations, deaths, and vaccination rates. Yet, data on preventative behavior is largely retrospective or unknown.

Our study addresses these shortcomings by presenting a novel way to measure individual-level behaviors in real time using geotagged social media images. The contribution is threefold. First, we develop an automated image classifier using a convolutional neural network (CNN) to detect images of people wearing masks and apply this classifier to geotagged Twitter data from March 1 through September 11, 2020. Twitter data were collected in real time and represent all publicly available (and approximately one-third of actual) geotagged tweets from the United States at this time. Second, we demonstrate that social media behavior closely tracks survey data using YouGov’s COVID-19 Public Monitor and Democracy Fund + UCLA Nationscape^7,8^. In doing so, we find that several individual-level correlates of mask wearing documented in observational research—age, gender, and partisanship—are also predictive of mask wearing on Twitter. Third, we investigate county-level factors that may impact widespread mask wearing. With 18,968,038 total geotagged tweets with images from 1,451,591 unique users, 73,489 of which posted mask wearing images, Twitter data are vast and allow us to examine less populated areas commonly underrepresented in surveys. We combine information on local mask wearing with county-level data on mask mandate policies, COVID-19 death rates, election returns, and individual mobility, as well as national media attention. While we find that a county’s 2016 GOP presidential vote share is negatively associated with mask wearing overall, the introduction of mask mandates in Republican-leaning counties is associated with larger increases in mask-wearing images than in counties that lean Democratic. National media attention and urbanization are also predictive of mask wearing, though local measures of population density and mobility are not.

## Benefits and drawbacks to using Twitter

This study highlights myriad advantages of using Twitter to measure the effect of political interventions on public health. First, since Twitter data are collected passively, they often lend themselves to long-term temporal estimates better than survey data. This feature is especially important for surprising events because it takes time to generate, test, and administer a survey. Twitter is able to generate estimates of mask wearing much earlier than surveys, even ones conducted online such as the U.S. COVID-19 Trends and Impact Survey (CTIS)^9^. Second, Twitter data enables the measurement of precise geographic areas. In this case, we focus on the county-level, where the majority of mask mandates took place. Third, survey respondents may misrepresent their true behaviors due to social desirability bias, a problem especially concerning for politically-sensitive behaviors. Since Twitter users do not think of themselves as survey respondents, measurements using the social media platform are less likely to exhibit bias. Last, surveys may not ask about mask wearing: CTIS, for example, did not start asking about masks until September 8, at which point mask mandates were already pervasive. The large, passively monitored, and less strategic behavior on Twitter compared to surveys has proved fruitful for a wide range of social and political studies, such as of crime, natural disasters, mass gatherings, and international conflict^10–16^.

At the same time, Twitter data have disadvantages stemming from non-representativeness and non-probability sampling. Surveys and academic studies show that Twitter users are not representative samples of the offline population^17–19^. Users must geotag a tweet in order for it to appear in this paper’s sample, introducing concerns about bias in users who employ this feature compared with those who do not. While these concerns are valid, it is worth noting that previous research finds Twitter users who share images without location information are not measurably different than those who share geotagged images^20^, suggesting that relying on users who geotag face mask images may not bias results compared to the larger Twitter population.

Because Twitter requires no identifying information for accounts, probability sampling is not feasible. This paper’s use of non-probability sampling is therefore problematic if the goal is to produce population estimates (Note: CTIS also collected data online via non-probability sampling using Facebook surveys.) We acknowledge and accept this limitation, given the fact that surveys do not provide sufficient geographic detail to measure the impacts of mask mandates. By applying computer vision techniques to a novel type of data, we have chosen a sampling design fit for this purpose^21^. However, the point estimates in this paper are most important for the trends they reveal and not as estimates of true rates of mask adherence.

## Materials and Methods

### Geotagged Twitter database

We compiled a dataset of geotagged publicly-available tweets from March 1 through September 11, 2020, collected in real time from the Twitter Application Programming Interface (API), using Twitter’s POST statuses/filter streaming endpoint. Approximately 3% of tweets in English are geocoded, and Twitter's API (v1.1) returns approximately 1/3 of all geotagged tweets^22,23^, resulting in 170,014,835 tweets from 2,603,654 U.S. users. Of these, 18,968,038 tweets contained an image, and 1,451,591 unique users posted at least one image.

Each geotagged tweet contains a global bounding box of coordinates identifying the tweet’s location. However, the size of the bounding box varies because users can specify one of five levels to geotag: country, state, city, neighborhood, or point of interest. We dropped all tweets with boxes above the city level (7% of tweets), because they were too large to provide county-level information. For cities that span multiple counties (14% of tweets that specify the city level), we assign the tweet to the county that covers the greatest share of the citys’ population^24^. All Twitter images analyzed in this study are publicly available and considered part of the public domain. (The use of public geotagged Twitter data was authorized by the University of California, Los Angeles Institutional Review Board, approval number #18-001354. All methods were performed in accordance with the UCLA IRB regulations and guidelines as well as the Declaration of Helsinki.)

### Mask detection

To automatically detect mask-wearing images, we developed an image classifier using a convolutional neural network (CNN) with the ResNet-50 architecture^25^. CNNs have been widely used for automated visual content analysis including facial mask detection^25–28^. We took a supervised learning approach to collect and annotate images with mask-wearing labels to train and evaluate our model. The quality of training data is critical to ensure the model’s performance. Therefore, we used an iterative approach to collect diverse and challenging data to make the final model robust.

To this end, we first collected 8000 images using Google’s image search API for three mask-related keyword phrases (‘wearing masks,’ ‘face covering,’ and ‘mask selfie’), as well as three keyword phrases not related to mask wearing (‘selfie,’ ‘hangout,’ and ‘stay at home’). Approximately 1300 images were collected for each phrase.

We then manually annotated each of the 8000 images as mask-wearing or non-mask-wearing. To be coded as mask-wearing, an image must show at least one human face occupying at least 5% of the image’s area wearing a medical or cloth mask over the mouth and nose. The rest of the images are negative samples that contain, for example, no face, faces without masks, faces occupying less than 5% of the image’s area, or faces in advertisements for masks. Such “hard” negatives samples present visuals similar to mask-wearing scenes and thus train a more robust classifier (See the work of Shrivastava et al.^29^, for example).

We use an active learning approach for robust model training that takes advantage of iterative model training and hard-data mining. Specifically, we first trained an initial model using the above dataset and applied it to a large set of unlabeled images, sourced from geotagged Twitter images from March 1–5, April 1–5, and May 20–25. Among these, 9391 images were classified positive. We manually verified their correctness and added the images and labels to the original set, resulting in 17,391 images. The optimal model parameters in the ResNet-50 classifier were obtained by stochastic gradient descent (SGD) with a binary cross entropy loss function, 500 training epochs, learning rate = 0.005, weight decay = 0.0004, and momentum = 0.9, as well as initial weights pre-trained on Imagenet data^30^. We did not include Twitter images predicted as negative by the initial classifier because there were so many of them, and the recall was very low. Finally, we randomly divided the annotated images in a training set (80%, 13,913 images) and a validation set (20%, 3478 images). These labeled images further fine tune the initial classifier. All manual labeling mentioned above was completed by one human coder. Since these annotations are not used for measurements in actual analysis, it is acceptable to have only one coder, as long as the model performance is satisfactory. Two other coders verified the main coder’s work by annotating a 1000-image random subset of the training set. The Krippendorff’s alphas between the primary coder and the two additional coders were 0.865 and 0.870, showing strong inter-rater reliability.

**Figure 1:**
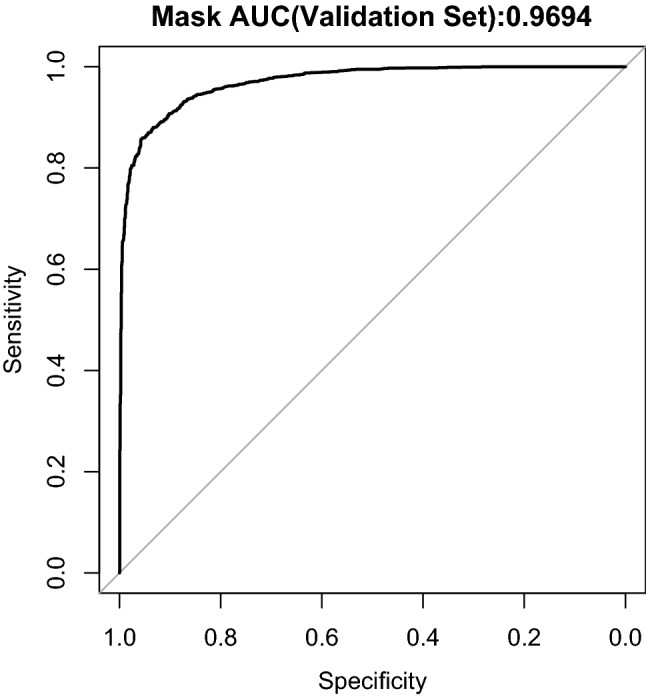
Receiver Operating Characteristics (ROC) curve for mask classifier on the validation set. Sensitivity is the fraction of actual positive data points that the model correctly classifies as positive. Specificity is the fraction of actual negative data points that the model correctly classifies as negative. Higher Area Under the Curve (AUC) values are associated with a greater ability to distinguish positive from negative examples.

Last, we chose an optimal decision threshold. Naively choosing 0.5 as the threshold may still yield an undesirable result (e.g., a too high false positive rate). Table 1 displays the classifier performance when using different decision thresholds on the same set. Since thresholds 0.7, 0.8, and 0.9 produce similarly high F1 scores (0.85, 0.86, and 0.85, respectively), we use a threshold of 0.9 in order to maximize precision (0.93). (Among all Twitter images, the proportion showing a mask is low. We manually verified a random subset of 1000 geotagged Twitter images posted on Aug 1, 2020 and found only 11 positive cases. To maximize our model precision in the face of potentially high false positives, we used the 0.9 threshold). With this threshold, the model has high classification accuracy. The model still demonstrates strong predictive power across other thresholds, as shown in the Receiver Operating Characteristic graph in Fig. 1.

**Table 1:** Validation accuracy of mask prediction model from images with different decision thresholds.

Threshold	Precision	Recall	F1 Score
0.50	0.77	0.87	0.82
0.60	0.81	0.85	0.83
0.70	0.86	0.83	0.85
0.80	0.92	0.81	0.86
0.90	0.93	0.77	0.85

Precision is calculated as the proportion of predicted mask images that actually show a mask. Recall is the proportion of actual mask images predicted as a mask image. F1 score = 2 $\times$ (Precision $\times$ Recall)/(Precision + Recall).

### Mask mandate coding

For the county-level analysis, the unit of observation is the county-week dyad, which covers a Monday-Sunday week. Each observation is coded as having a mask mandate in place if a mandate started on or before the Thursday in that week.

### Case-control sampling

For the individual-level analyses, we perform a case-controlled sampling design by collecting all tweets with mask-wearing images, as well as a random sample of 1000 tweets per day from the set of non-mask images^31^. Then we extract demographic features of users who posted these tweets to calculate odds ratios.

### Sample size determination

The Twitter data is imbalanced across geographical regions. Table 2 displays key summary statistics for the weekly number of Twitter images and users who posted images in the geotagged Twitter dataset. In some less populated counties, the sample size become too small for our analysis to have sufficient statistical power. To determine the number of tweets with images needed per county, the following calculation is performed. The null and alternative hypothesis proportions are $p_{\mu } = 0.005$ and $p_{\alpha } = 0.02$, respectively, which are equal to the approximate minimum ($p_{\mu }$) and maximum ($p_{\alpha }$) values of the nationally aggregated mask wearing on Twitter. Let the level of significance $\alpha = 0.05$ and the desired power = $1- \beta$ = 0.9, where $\beta$ = 0.1 is the probability of committing a Type II error. Using Cohen’s arcsine transformation^32^, the effect size is $h = 2 * (\arcsin {\sqrt{p_{\alpha }}} - \arcsin {\sqrt{p_{\mu }}}) = 0.142$. Under the null hypothesis, $\mu _0$, the decision boundary ${\hat{p}}$ should have z-score $Z_0$, where $P(Z<Z_0) = 1-\alpha \div 2 = 0.975$ for $Z \sim N(0,1)$, so $Z_0$ = 1.959. Under the alternative hypothesis, $\mu _a$, $\bar{X}$ should have a z-score $Z_a$, where $P(Z<Z_a) = \beta = 0.1$, so $Z_a$ = − 1.281. Thus the required sample size $n = \left( {\frac{{Z_{0} - Z_{a} }}{h}} \right)^{2} = 519$. In our sample, 463 of 3006 counties have at least 519 unique Twitter users with images in the entire collected geotagged dataset. These counties account for 77% of the U.S. population.

**Table 2:** Weekly number of users who posted images/mask-wearing images by county.

	Min	Median	Mean	Q3	95% percentile	Max	Std
Image users	1	16	110.19	60	443	16,410	450.8
Mask users	0	0	1.98	1	8	343	9.2

## Results

### Validating Twitter image data using national surveys

To assess how well Twitter image data captures mask-wearing trends across the nation, we compare aggregate levels of mask wearing in our sample with self-reports from two nationally-representative surveys run during this period: YouGov’s COVID-19 Public Monitor^7^ and UCLA Nationscape^8^. These online surveys asked the public whether or not they wore a mask in public spaces during the past week. (YouGov’s survey stated: “Thinking about the last 7 days, how often have you: worn a face mask outside your home to protect yourself or others from coronavirus (COVID-19)?” Response options included “always,” “frequently,” “sometimes,” “rarely,” and “not at all.” The UCLA Nationscape survey asked respondents “Have you done any of the following in the past week?” with “Worn a mask when going out in public” as one of the possible categories and a binary response option of “Yes” or “No.”) The Twitter measure is the fraction of users posting any image that also posted a mask image over a given week.

**Figure 2:**
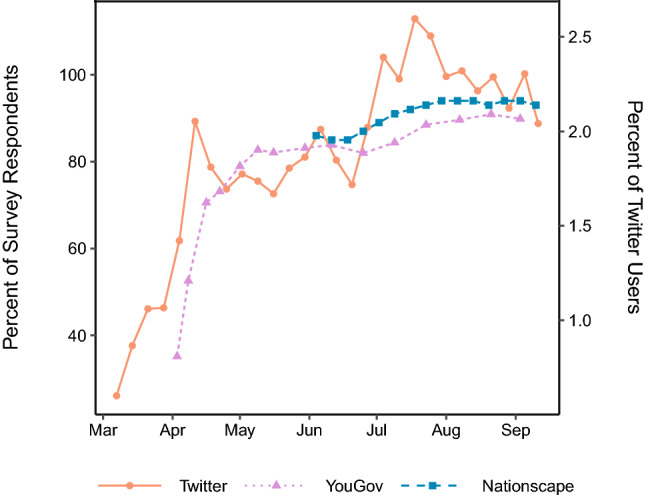
Mask wearing from March through September, 2020. The left vertical axis shows percentage of survey respondents who reported wearing a mask in public. The right vertical axis shows the percentage of Twitter users who posted a mask image (among those who posted at least one image in a geotagged tweet).

Figure 2 reveals a strong association between the estimates using Twitter images and both surveys. (Note the dual verticle axes to accommodate different magnitudes from surveys and Twitter.) Both Twitter and YouGov reveal similar increases in public mask wearing from April to May. (Nationscape did not ask about mask wearing for the first half of the data.) All estimates increased at a similar level from June to September. High correlations between mask usage on Twitter and self-reports from YouGov ($r=0.61, p<0.01, N = 18$) and Nationscape ($r=0.79, p<0.01, N=10$) suggest that social media images are highly predictive of similar behaviors offline. (In addition, the Twitter data correlate with a different YouGov study running from March through September 2020 at $r=0.84$. We do not include this study in Fig. 2 because the individual-level data is not publicly available^33^.) We also took the first differences of time series to address non-stationarity. The resulting stationary Twitter time series is still significantly correlated with YouGov ($r=0.63, p<0.01, N = 17$) and Nationscape ($r=0.80, p<0.01, N=9$). (We present correlations in the time-series by state in Supplementary Information Section 6.) County-level data allows for more precise estimates of how mask mandates and partisanship interact. (The only survey we know of that identifies mask wearing metrics at the county level is one conducted by *The New York Times* in July of 2020^34^. That survey, however, only lasted for a 12-day period (July 2–July 14) and thus did not facilitate the same observational study overtime.)

### Individual-level determinants

Previous research examining individual-level factors finds that older individuals are more likely to wear masks than their younger counterparts^8,34–37^ (although see the work of MacIntyre et al.^38^ and Howard^39^). Females are also more likely than males to wear a mask^36,39–42^, and Democrats are consistently more likely than Republicans to not only to wear a mask, but to support mask mandates^39–42^. To test the validity of our data, we compare mask wearing across age, gender, and political ideology for Twitter users. A user’s age and gender are estimated by applying a deep neural network facial classifier trained by FairFace^43^ on the user’s profile image. *Conservative Leaning* measures a user’s ideology based on the number of influential partisan-leaning Twitter accounts that individual follows. Users with a *Conservative Leaning* score greater than 0 are generally considered Republicans^44^. Previous research identifies partisanship as the strongest predictor of mask use^45^.

Table S4 reports odds ratios for each demographic group in relation to the specified reference group. In line with previous research, we find that seniors, females, and Democrats demonstrate significantly higher likelihoods of mask wearing. (FairFace is also capable of classifying race, but the accuracy on Twitter profiles is unsatisfactory—64% on a random sample of 1000 profiles. The odds ratios between race groups classified by FairFace are not significant in our data.)

Similar to previous studies which reported yield rates when analyzing Twitter users^44,46^, we are able to infer gender and age from 20.4% of users and partisan leaning from 39.4% of users. Accuracy rates for the algorithms we use that assign socio-demographic and political-leaning characteristics are extremely high. Previous work finds that the average classification accuracy for the algorithms we use is 94.4% for gender and 60.2% for age^43^. Scholars have validated the Twitter partisanship measure employed here by matching it with campaign contribution records using name and zip code. The correlation between campaign contributions and political leaning is also very high (r = 0.80)^44^. To provide further evidence that our partisanship estimates match the actual population, we correlated Republican vote share in the 2016 election with the share of Twitter users we infer to be Republicans—among those for whom we can infer political leaning—and found they are highly correlated. Across all weeks, they correlate at $0.63 (N=100, p < 0.01)$. Across all counties, the correlation is $0.29, N = 3009, p < 0.01$.

Many studies find that the inferred socio-demographic and political leaning variables are supported in survey data. The canonical article about Twitter and ideology^44^ shows that the latter is recoverable from follower relationships on Twitter, and that this measure sorts accounts accurately^47^. Comparing survey respondents’ self-described Twitter behaviors with their actual Twitter behavior reveals that the two are positively correlated^47^. Other work^48^ connects Twitter users with voter files and shows that the demographics and ideology of Twitter users match voting administrative data. And, scholars have successfully estimated protesters’ age and gender using Twitter images with surveys of actual protesters^15^.

### Contextual determinants: mask mandates, mobility, death rates, and county partisanship

To examine contextual factors that shape mask wearing, we calculate the percent of users who post at least one mask image (among those who post any image) for each week in the data. By only including users who post at least one image of any kind, we control for unobserved heterogeneity that may reflect differences between users who share images and those who do not.

**Figure 3:**
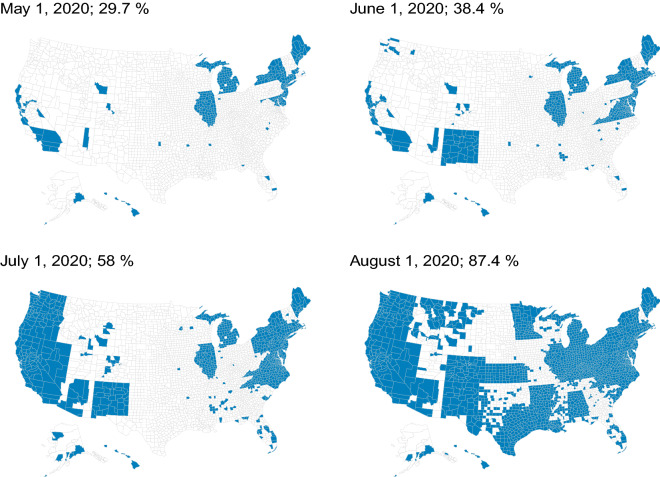
Counties under a mask mandate. Percentages indicate the share of the U.S. population under a mask mandate on the corresponding date. Mandate data are acquired from Wright et al.^49^.

Mask mandate data come from Wright et al.^49^. This database includes the earliest effective dates of local and state mask mandates for every U.S county and runs through August 4, 2020. Figure 3 shows the share and distribution of counties, as well as the corresponding share of the U.S. population, under an active county or state mandate (or both) from May 1 through August 1, 2020. As of August 1, 2020, 66.3% of U.S counties, and 87.4% of the U.S. population were under active mask-wearing mandates.

Table 3 presents summary statistics for each of the continuous explanatory variables of interest. Subscript *i* denotes different counties, *t* denotes different weeks, and $t-1$ indicates a lag of one week. In selecting our control variables, we have attempted to address many of the factors that might plausibly be associated with variations in the incidence or severity of COVID-19, or with the likely local or personal response to the pandemic. For example, greater news coverage of the pandemic or a spike in the number of deaths might reasonably increase the probability an individual would choose to wear a mask, regardless of whether there is a governmental requirement to do so. Close proximity to others due to geography or the activity of individuals might also be expected to increase the precautions one might take against contracting COVID-19. Finally, there have been clear and ongoing partisan differences related to mask-wearing throughout the pandemic, as illustrated in various prior studies and surveys^8,42,45^.

*Deaths per 10k* is the number of reported COVID-19 deaths in the county during the prior week per 10,000 residents^50^. *GOP Vote 2016* is the share of the two-party vote that went to Donald Trump in the 2016 presidential election^51^. *Urban Population Percentage*^52^ is the percent of a county’s population living in a Census-defined urban area. *Population Density*^53^ is the county’s population (using 2010 Census data) divided by its area in square miles. Media coverage of the pandemic may drive public attention to COVID-19 and increase mask wearing. *COVID News* measures the average number of times the word “COVID” is used per national morning or evening news show on ABC, CBS, and NBC^54^. Last, images of mask wearing may decrease as people venture outside their homes. We use Safegraph’s Places of Interest dataset to measure changes in activity^55^. Specifically, *Retail Visit per Hundred* measures the number of visits to a grocery store, as defined by those with NAICS business codes 44 or 45, in a county during the current week per 100 residents. In addition, the following models include a *Week Counter*, which ranges from 1 to 22 and controls for unobserved time trends. See Table S7 for models with different subsets of control variables.

**Table 3:** Explanatory Variables 95% Intervals for the 300 most populous counties and all U.S. counties.

	Top 300 counties	All counties
Deaths per 10$\hbox {k}_{i, t-1}$	0, 1.6	0, 1.0
GOP vote 2016$_i$	15.3, 70.1	23.7, 85.1
Population $\hbox {Density}_i$	81.7, 11,379.5	5.2, 2630.0
COVID $\hbox {News}_t$	0.9, 11	0.9, 11
Retail visit per $\hbox {Hundred}_{i, t}$	0.67, 4.24	0.61, 5.58

Like the population, tweets are imbalanced across counties. Although Twitter data generally produces samples larger than surveys in less populated areas, the sample sizes in the majority of the country’s 3,006 counties are still too small to use for a powerful statistical test (See Table S5 and S6 for sample sizes and numbers of Twitter users/survey respondents wearing masks). As a result, the main regression is run for the 300 most populous counties, which together account for approximately 64% of the United States population. All of the top 300 counties have more than 519 unique image-posting users, thus satisfying the sample size calculated in the Sample Size Determination section. There are no substantive differences found when we run the regression on the top 100, top 500, or all counties, as shown in Table S1.

**Table 4:** Contextual predictors of mask-wearing images. The dependent variable is the proportion of Twitter users who posted mask-wearing images among users that posted any tweets with images.

Variable	No interaction	With interaction
	Estimate(SE)	Estimate(SE)
Intercept	$$\mathbf {-5.129}{***}$$ (0.257)	$$\mathbf {-5.065}{***}$$ (0.256)
Mask mandate	$$\mathbf {0.058}{***}$$ (0.014)	$$\mathbf {-0.055}{*}$$ (0.028)
GOP vote 2016	$$\mathbf {-0.005}{***}$$ (0.001)	$$\mathbf {-0.006}{***}$$ (0.001)
Mask mandate * GOP vote 2016		$$\mathbf {0.003}{***}$$ (0.001)
Deaths per 10k	0.014 (0.007)	0.011 (0.007)
Urban population percentage	$\mathbf {0.004}{*}$ (0.002)	$\mathbf {0.004}{*}$ (0.002)
Population density	0.000 (0.000)	0.000 (0.000)
COVID News	$$\mathbf {0.066}{***}$$ (0.014)	$$\mathbf {0.064}{***}$$ (0.014)
Retail visit per hundred	0.021 (0.016)	0.008 (0.016)
Week counter	$$\mathbf {0.028}{***}$$ (0.006)	$$\mathbf {0.029}{***}$$ (0.006)
AIC	31989.304	31967.327
BIC	32070.837	32055.653
Log likelihood	$-15982.652$	$-15970.663$
County weeks	6600	6600
States (Including D.C)	45	45
Num. groups: week	22	22
County intercept	0.038	0.037
State intercept	0.014	0.014
Week intercept	0.027	0.027

$${***}p<0.001$$; $${**}p<0.01$$; $${*}p<0.05$$

Table 4 presents results from two multilevel generalized linear models^56^ in the binomial family with random effects for week, county, and state. (A generalized linear model with a binomial family is ideal for modeling proportions that are otherwise prone to heteroskedasticity while accommodating lower and upper bounds at 0 and 1.) The first model examines the effect of *Mask Mandate* and *GOP Vote 2016* without an interaction effect. As we can see the mandate is is positive and significant; overall, people are more likely to wear a mask when there is a mandate in place. Republican support is, as expected, negative and significant. More conservative counties on the whole see fewer individuals posting Twitter images with masks.

Now let us turn to the interaction model. (Because we include an interaction between *Mask Mandate* and *GOP Vote 2016*, we must interpret the effects of the two variables jointly. For example, the negative coefficient on *Mask Mandate* in the interaction model can only be interpreted for a county with zero percent GOP vote, which is never the case.) Under no mask mandate, county-level Republican leaning shows a negative, significant association with mask wearing: for a one standard deviation increase (12.8 percentage points) in GOP vote share in the 2016 presidential election, the expected level of mask wearing decreases 7.4 percent. Not surprisingly, mask wearing is lower in Republican strongholds, all else equal. This negative association is consistent with previous findings^45^.

**Figure 4:**
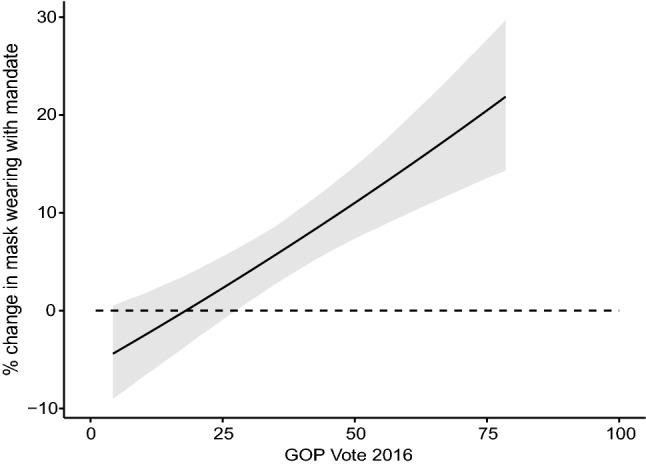
Marginal Effect of Mask Mandates for Varying GOP County Vote. Shaded areas represent 95% confidence intervals.

The introduction of a mandate, however, is strongly associated with an increase in mask wearing. Moreover, this association is especially large in counties that favored Donald Trump in 2016. To illustrate these interactive effects, Fig. 4 shows the predicted marginal effect of a mandate for varying levels of GOP vote share. Moving from the county with the lowest percentage vote for the GOP to the county with the highest vote increases the expected effect of mandates by 25 percentage points. Although mask mandates have a greater predicted effect in Republican counties, the overall level of mask wearing remains slightly higher in Democratic counties. Indeed, Republican counties with mask mandates display similar levels of mask wearing to Democratic counties without mandates. These results suggest that in majority-Democratic counties individuals are more likely to wear a mask regardless of whether there is a legal mandate requiring them to do so or not, whereas in majority-Republican counties mandates may prompt people to put on a mask, thus narrowing the gap caused by partisanship. (Using a binned estimator^57^ to evaluate flexible marginal effects produces similar results.)

News coverage is positively associated with mask wearing images: when the average occurrence of the word “COVID” increases by one standard deviation (2.9 times), mask wearing is predicted to increase by 20.7%–21.4% across the models. To a large extent, the news variable mirrors what people are tweeting about. In particular, COVID-19 news decreased by approximately 60% from week 13 to week 16 during the protests following George Floyd‘s death, and mask wearing images decreased during this time as well. (Table S2 shows the same regression models using cable rather than network news coverage and finds the same positive and significant association with mask wearing.)

Mask wearing also increases with urbanization: A one standard deviation rise in urbanization (25 percentage points) corresponds to a $10.5\%$ increase in mask images. The remaining population variables - deaths and density - are not statistically significant in most models. Mask-wearing images also do not decrease with higher rates of grocery store visits. Not surprisingly, the positive coefficient on the linear week counter demonstrates an overall increasing trend in public mask wearing. As time goes by, mask wearing becomes a habit for many individuals. Table S3 shows the estimation with a logged week counter and demonstrates the same effect of time.

Respondents in Nationscape are identified at both the state and the congressional district level, while respondents in YouGov are only identified at the state level. Thus, we performed another check of the hypotheses at the individual level focusing only on Nationscape respondents. The dependent variable indicates whether or not the person stated that they wore a mask in the previous week. The key independent variables are respondent Party ID (ranging from 1 = “strong Democrat” to 7 = “strong Republican”), Mask Mandate (indicating if there was a mandate in that congressional district in the week prior to the survey), and their interaction. We controlled for individual demographic variables including age, gender, ethnicity, household income, and education level. We also controlled for district-level features including population density and urban population proportion. As in the county-level analysis, we included a week counter to control for a general time trend, and national level COVID News variables. The regression output is reported in Table S8 in the Supplementary Information. We found the same trend as indicated on the county-level analyses: while higher GOP partisanship itself is correlated with lower mask wearing, the interaction of GOP partisanship and mask mandate is positive and significant, indicating that Republicans may be more likely to change their behavior during mask mandates than Democrats.

## Discussion

Using Twitter, and digital trace data more broadly, confers a number of benefits. In contrast with surveys, Twitter data do not rely on retrospective responses, which often suffer due to memory loss and social desirability biases. Twitter data provide a measure that is free and can be collected and stored on demand in real time. The new Academic Search Product provides any published tweet that is still public, facilitating post hoc studies. In addition, the sheer volume of Twitter data allows researchers to estimate effects across groups that would otherwise be prohibitively costly. Though Twitter is not a representative sample of Americans^19^, it can serve as a meaningful thermometer for public opinion and health behavior, despite sometimes drastically different levels of measurement^14^.

Moreover, social media image data provide an invaluable and unobtrusive way to measure public health behaviors and trends^58,59^. To our knowledge, this is among the earliest work to collect COVID-19 Twitter image data and apply deep learning based image classification^60^, and the first to measure public health behaviors related to COVID-19 with such methods. Future research may employ social media images to identify other behaviors or attributes, such as smoking, alcohol or drug use, obesity, and seat-belt compliance.

Despite the advantages of using geotagged tweets to study public health behavior, there are drawbacks. One difficulty is class imbalance across outcomes (there are many more images without facemasks than with them), which could cause a classifier to fit out of sample data poorly^61^. We avoid this problem by constructing training datasets with an equal number of facemask and non-facemask images and later choose a classification threshold high enough to generate a precision of 0.93. A second drawback concerns the potential bias in user behavior during this time period. Given that people likely stay home more during mask mandates, they may also tweet less or remove masks when taking photos. Either behavior could cause us to underestimate changes in mask wearing. Future research may incorporate the content of tweets to better identify a person’s location or to ascertain information about other user characteristics of interest, such as education or income. Because most geotagged tweets are only located at the level of neighborhoods or larger geographical regions, our research was unable to identify whether or not photos are taken in public settings. “Selfie” images taken indoors do not provide sufficient information to distinguish between public and private locations. (We also considered surveillance camera video data as an alternative indicator of mask-wearing behavior. Unfortunately, these data simply do not exist in a way that provides the coverage necessary for this study. Publicly-available surveillance data rely on footage from security cameras that use non-password-protected internet connections. Putting the ethical issues aside, these feeds tend to not be taken in environments that are conducive to the study, such as residential buildings rather than commercial settings.)

The principle finding that mandates are more closely associated with mask wearing in areas with more Republican voters suggests that governmental policies have the ability to equalize health behaviors, and potentially health outcomes. We know that conservative voters are less likely to vaccinate against COVID-19^62^, and early research suggests that vaccine mandates are effective at increasing compliance^63,64^. Although systematic experimental analyses have yet to be conducted, our study suggests that vaccine mandates may have had the greatest effect in GOP strongholds.

Last, it is worth noting that Twitter postings are not simply a random slice of life, but might also reflect individuals’ strategic choices aimed at influencing friends or public opinion. These decisions likely change over time in response to the prevalence and politicization of mask wearing, and future research would benefit from exploring a dynamic relationship between mask images, mandates, and the political climate.

## Supplementary Information

Supplementary Information.

## Data Availability

All aggregate-level data, deidentified individual-level data, and replication files are available on Dataverse doi:10.7910/DVN/1ZBK71. The image classifier code and trained model are available through the Github repository https://github.com/Bernardo1998/Mask-Wearing-in-Geolocatted-Twitter.
