# Supplementary material for: Mask images on Twitter increase during COVID-19 mandates, especially in Republican counties
Source: Sci Rep. 2022 Dec 9;12:21331. doi: 10.1038/s41598-022-23368-6 (PMC9734103; doi:10.1038/s41598-022-23368-6)
Supplement: Supplementary file 1 — Supplementary Information. [file 41598_2022_23368_MOESM1_ESM.pdf]

Supplementary Information

1 Regression On Different County Subsets

	Top 100 Counties	Top 500 Counties	All Counties
Variable	Estimate(SE)	Estimate(SE)	Estimate(SE)
(Intercept)	-5.479^{***}	-5.028^{***}	-4.800^{***}
	(0.683)	(0.182)	(0.129)
Mask Mandate	0.002	-0.073^{**}	-0.085^{***}
	(0.032)	(0.026)	(0.024)
GOP Vote 2016	-0.005^{*}	-0.006^{***}	-0.005^{***}
	(0.002)	(0.001)	(0.001)
Mask Mandate * GOP Vote 2016	0.002^{**}	0.003^{***}	0.003^{***}
	(0.001)	(0.001)	(0.001)
Deaths per 10k	0.010	0.013	0.015^{*}
	(0.008)	(0.007)	(0.007)
Urban Population Percentage	0.007	0.005^{***}	0.001^{*}
	(0.007)	(0.001)	(0.001)
Population Density	0.000	0.000	0.000^{*}
	(0.000)	(0.000)	(0.000)
COVID News	0.068^{***}	0.060^{***}	0.059^{***}
	(0.015)	(0.014)	(0.013)
Retail Visit per Hundred	0.056^{*}	-0.021	-0.032^{***}
	(0.025)	(0.013)	(0.008)
Week Counter	0.025^{***}	0.031^{***}	0.032^{***}
	(0.007)	(0.006)	(0.006)
AIC	14838.811	43294.338	67920.796
BIC	14912.861	43389.312	68031.563
Log Likelihood	-7406.405	-21634.169	-33947.398
County-Weeks	2200	11000	37070
Counties	100	500	1685
States (Including D.C)	31	50	51
County Intercept	0.024	0.043	0.052
State Intercept	0.008	0.017	0.011
Week Intercept	0.030	0.025	0.024

$^{***}p < 0.001$; $^{**}p < 0.01$; $^{*}p < 0.05$

Table S1. Contextual effects of mask-wearing images.

2 County Level Regression with COVID News from FOX/CNN/MSNBC

	Top 100 Counties	Top 300 Counties	Top 500 Counties
Variable	Estimate(SE)	Estimate(SE)	Estimate(SE)
Intercept	$\mathbf{-5.512}^{***}$	$\mathbf{-5.075}^{***}$	$\mathbf{-5.036}^{***}$
	(0.683)	(0.256)	(0.182)
Mask Mandate	0.005	−0.052	$\mathbf{-0.069}^{**}$
	(0.032)	(0.027)	(0.026)
GOP Vote 2016	$\mathbf{-0.005}^{*}$	$\mathbf{-0.006}^{***}$	$\mathbf{-0.006}^{***}$
	(0.002)	(0.001)	(0.001)
Mask Mandate * GOP Vote 2016	$\mathbf{0.002}^{**}$	$\mathbf{0.003}^{***}$	$\mathbf{0.003}^{***}$
	(0.001)	(0.001)	(0.001)
Deaths per 10k	0.010	0.011	0.013
	(0.008)	(0.007)	(0.007)
Urban Population Percentage	0.008	$\mathbf{0.004}^{*}$	$\mathbf{0.005}^{***}$
	(0.007)	(0.002)	(0.001)
Population Density	0.000	0.000	0.000
	(0.000)	(0.000)	(0.000)
COVID News (Cable)	$\mathbf{0.105}^{***}$	$\mathbf{0.100}^{***}$	$\mathbf{0.093}^{***}$
	(0.023)	(0.022)	(0.021)
Retail Visit per Hundred	$\mathbf{0.057}^{*}$	0.008	−0.021
	(0.025)	(0.016)	(0.013)
Week Counter	$\mathbf{0.026}^{***}$	$\mathbf{0.029}^{***}$	$\mathbf{0.031}^{***}$
	(0.007)	(0.006)	(0.006)
AIC	14879.494	32526.891	43409.854
BIC	14953.545	32615.224	43504.828
Log Likelihood	−7426.747	−16250.446	−21691.927
County-Weeks	2200	6600	11000
States (Including D.C)	31	45	50
County Intercept	0.024	0.037	0.043
State Intercept	0.008	0.015	0.018
Week Intercept	0.030	0.027	0.025

$^{***}p < 0.001$; $^{**}p < 0.01$; $^{*}p < 0.05$

Table S2. Contextual effects of mask-wearing images. *COVID News* in this model only includes data from FOX/CNN/MSNBC.

3 County Level Regression Using Logged Time

	Top 100 Counties	Top 300 Counties	Top 500 Counties
Variable	Estimate(SE)	Estimate(SE)	Estimate(SE)
Intercept	**−5.652******	**−5.221******	**−5.193******
	(0.679)	(0.248)	(0.171)
Mask Mandate	0.000	**−0.054***	**−0.072****
	(0.032)	(0.027)	(0.026)
GOP Vote 2016	**−0.004***	**−0.006*****	**−0.006*****
	(0.002)	(0.001)	(0.001)
Mask Mandate * GOP Vote 2016	**0.002****	**0.003*****	**0.003*****
	(0.001)	(0.001)	(0.001)
Deaths per 10k	0.010	0.011	0.013
	(0.008)	(0.007)	(0.007)
Urban Population Percentage	0.008	**0.004***	**0.005*****
	(0.007)	(0.002)	(0.001)
Population Density	0.000	0.000	0.000
	(0.000)	(0.000)	(0.000)
COVID News	**0.042****	**0.038****	**0.034****
	(0.013)	(0.012)	(0.011)
Retail Visit per Hundred	**0.053***	0.006	−0.022
	(0.025)	(0.016)	(0.013)
Week Counter Log	**0.292*****	**0.318*****	**0.329*****
	(0.045)	(0.042)	(0.039)
AIC	14822.656	32422.481	43279.280
BIC	14896.707	32510.814	43374.253
Log Likelihood	−7398.328	−16198.241	−21626.640
County-Weeks	2200	6600	11000
States (Including D.C)	31	45	50
County Intercept	0.024	0.038	0.043
State Intercept	0.008	0.015	0.017
Week Intercept	0.017	0.014	0.013

$^{***}p < 0.001$; $^{**}p < 0.01$; $^{*}p < 0.05$

Table S3. Contextual effects of mask-wearing images, with a logged week counter.

4 Odds Ratios of Mask-wearing

Variable	Odds Ratio	95% CI
Age: 30-49	1.57	(1.49, 1.66)
Age: 50 plus	2.14	(1.99, 2.30)
Female	1.35	(1.28, 1.43)
Democrat	1.65	(1.60, 1.70)
N (Ages and Genders)		35321
N (Partisanship)		68076

Table S4. Odds Ratios. Odds ratios for each demographic group against the corresponding reference group (age 20-29. male, Republican leaning). The numbers of missing values in profiles and ideological leaning are different, resulting in different sample sizes.

5 LASSO Regression

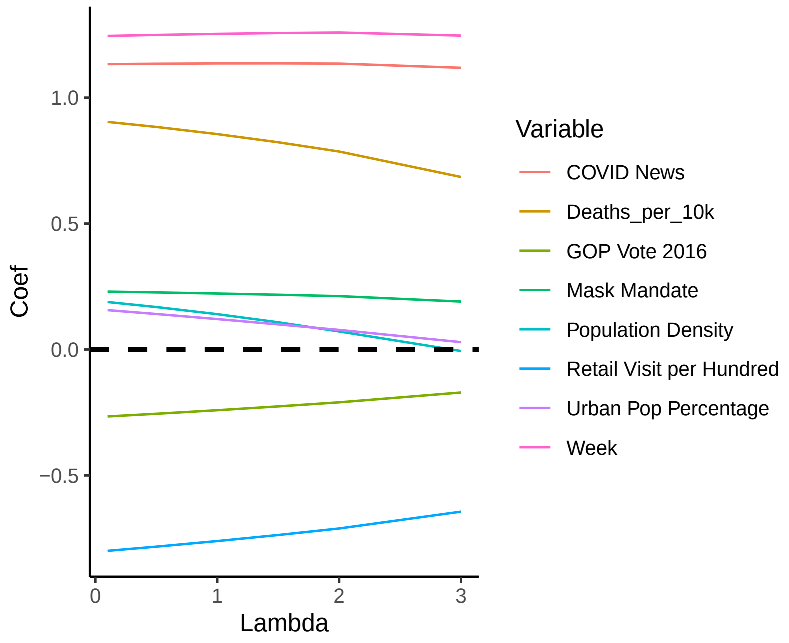

Fig S1. Coefficient estimates of predictor variables at different λ values.

6 State Level Correlation

State	Average Weekly Image Users/Respondents		Correlation
	YouGov	Twitter	
New York	59.11	3462.05	0.88
Texas	50.61	6245.45	0.85
North Carolina	19.28	1478.68	0.84
California	72.61	8045.59	0.82
Michigan	18.61	1137.27	0.81
Massachusetts	14.39	1079.59	0.80
Washington	15.06	1049.50	0.78
Missouri	12.94	762.95	0.78
Tennessee	14.56	1176.64	0.76
Florida	54.06	3950.36	0.75
Pennsylvania	35.89	1514.82	0.72
Ohio	26.83	1752.59	0.71
Alabama	13.06	643.45	0.70
New Jersey	23.11	1503.86	0.69
Wisconsin	13.44	500.27	0.69
South Carolina	11.89	668.64	0.69
Illinois	25.72	1997.68	0.68
Maryland	11.72	1103.77	0.68
Virginia	20.17	1226.95	0.68
Louisiana	6.33	905.64	0.67
Georgia	23.17	1848.59	0.65
Iowa	5.33	385.14	0.64
Indiana	13.83	975.18	0.62
Connecticut	7.33	452.27	0.61
Mississippi	4.78	332.95	0.60
Kentucky	12.11	513.95	0.59
Nevada	8.22	989.77	0.54
Oregon	11.11	603.32	0.52
Utah	5.61	399.68	0.52
Kansas	5.17	409.91	0.50
Arizona	17.33	1189.45	0.49
Colorado	12.06	868.77	0.49
Minnesota	12.17	666.18	0.49
Arkansas	5.89	294.00	0.46
Oklahoma	6.78	556.00	0.45
Rhode Island	1.83	182.36	0.43
Alaska	3.06	95.45	0.42
Hawaii	2.50	264.41	0.41
Montana	2.83	83.41	0.40
District of Columbia	2.89	599.55	0.38
New Hampshire	3.89	134.86	0.35
New Mexico	4.11	250.36	0.33
West Virginia	4.83	169.77	0.28
Nebraska	3.89	300.23	0.26
Delaware	2.61	97.36	0.19
Maine	3.33	112.64	0.18
Idaho	4.56	156.36	0.13
Vermont	1.50	46.82	0.03
North Dakota	0.94	77.55	0.01
South Dakota	1.44	99.91	-0.01
Wyoming	1.83	72.18	-0.04

Table S5. Pearson's correlation of state level time series between the YouGov survey and Twitter metric.

State	Average Weekly Image Users/Respondents		Correlation
	Nationscape	Twitter	
Oregon	80.80	603.32	0.93
Texas	452.00	6245.45	0.89
Tennessee	122.00	1176.64	0.88
Oklahoma	65.70	556.00	0.88
New Jersey	166.70	1503.86	0.82
Indiana	130.80	975.18	0.82
Louisiana	69.10	905.64	0.80
Montana	15.20	83.41	0.76
Minnesota	86.00	666.18	0.75
Kentucky	89.10	513.95	0.73
Nevada	71.70	989.77	0.71
Florida	462.70	3950.36	0.68
Illinois	268.50	1997.68	0.67
Kansas	56.00	409.91	0.66
Missouri	129.50	762.95	0.64
Virginia	148.70	1226.95	0.63
Arizona	148.90	1189.45	0.62
West Virginia	37.50	169.77	0.61
South Dakota	11.80	99.91	0.58
Ohio	261.90	1752.59	0.56
Alabama	87.30	643.45	0.53
Washington	128.30	1049.50	0.51
Michigan	170.20	1137.27	0.50
South Carolina	95.70	668.64	0.50
Utah	45.90	399.68	0.46
Wyoming	10.30	72.18	0.44
Pennsylvania	247.20	1514.82	0.43
Idaho	27.30	156.36	0.40
California	621.80	8045.59	0.38
Nebraska	34.00	300.23	0.38
Colorado	95.50	868.77	0.37
Alaska	8.90	95.45	0.36
Wisconsin	115.40	500.27	0.33
Mississippi	48.00	332.95	0.32
Maine	24.90	112.64	0.25
Arkansas	50.30	294.00	0.25
New York	390.20	3462.05	0.24
Iowa	57.30	385.14	0.22
Georgia	185.00	1848.59	0.16
Hawaii	25.50	264.41	0.12
Connecticut	58.00	452.27	0.05
North Carolina	181.90	1478.68	0.05
North Dakota	11.50	77.55	0.01
Rhode Island	13.80	182.36	-0.08
Maryland	99.90	1103.77	-0.10
New Hampshire	16.90	134.86	-0.12
Vermont	10.00	46.82	-0.13
Massachusetts	98.80	1079.59	-0.14
New Mexico	34.70	250.36	-0.28
District of Columbia	12.60	599.55	-0.34
Delaware	21.00	97.36	-0.50

Table S6. Pearson's correlation of state level time series between the Nationscape survey and Twitter metric.

7 Verifying Control Variables

	No Death	No Visit	No News	No News or Visit
Variable	Estimate(SE)	Estimate(SE)	Estimate(SE)	Estimate(SE)
Intercept	**−5.075*****	**−5.045*****	**−4.715*****	**−4.707*****
	(0.256)	(0.252)	(0.253)	(0.251)
Mask Mandate	**−0.056***	**−0.058***	**−0.057***	**−0.058***
	(0.028)	(0.027)	(0.028)	(0.027)
GOP Vote 2016	**−0.006*****	**−0.006*****	**−0.006*****	**−0.006*****
	(0.001)	(0.001)	(0.001)	(0.001)
Mask Mandate * GOP Vote 2016	**0.003*****	**0.003*****	**0.003*****	**0.003*****
	(0.001)	(0.001)	(0.001)	(0.001)
Deaths per 10k		0.011	0.011	0.011
		(0.007)	(0.007)	(0.007)
Urban Population Percentage	**0.004***	**0.004***	**0.004***	**0.004***
	(0.002)	(0.002)	(0.002)	(0.002)
Population Density	0.000	0.000	0.000	0.000
	(0.000)	(0.000)	(0.000)	(0.000)
COVID News	**0.065*****	**0.063*****		
	(0.014)	(0.014)		
Retail Visit per Hundred	0.010		0.005	
	(0.016)		(0.016)	
Week Counter	**0.029*****	**0.029*****	**0.042*****	**0.042*****
	(0.006)	(0.006)	(0.008)	(0.008)
AIC	32437.853	32435.951	32450.511	32448.607
BIC	32519.391	32517.489	32532.048	32523.350
Log Likelihood	−16206.926	−16205.975	−16213.255	−16213.303
County-Weeks	6600	6600	6600	6600
Counties	300	300	300	300
States	45	45	45	45
Weeks	22	22	22	22
County Intercept	0.037	0.037	0.037	0.037
State Intercept	0.014	0.014	0.014	0.014
Week Intercept	0.028	0.027	0.054	0.053

$^{***}p < 0.001$; $^{**}p < 0.01$; $^{*}p < 0.05$

Table S7. Regression models on the top 300 counties with different subsets of control variables. The inclusion and removal of COVID News and Retail Visit do not substantively change the results.

8 Individual Regression using Nationscape Survey Data

	Nationscape Respondents
Variable	Estimate(SE)
Intercept	-0.147
	(0.328)
Mask Mandate	-0.010
	(0.092)
Party ID	$\mathbf{-0.198}^{***}$
	(0.009)
Mask Mandate * Party ID	$\mathbf{0.056}^{***}$
	(0.016)
Week Counter	$\mathbf{0.125}^{***}$
	(0.010)
Age	$\mathbf{0.014}^{***}$
	(0.001)
Male	$\mathbf{-0.288}^{***}$
	(0.030)
Urban Population Proportion	$\mathbf{1.253}^{***}$
	(0.107)
Population Density	0.000
	(0.000)
COVID News	$\mathbf{0.039}^{*}$
	(0.019)
AIC	30572.560
BIC	31099.747
Log Likelihood	-15226.280
Respondents	48361
Congressional Districts	438
States	35
Weeks	11
Congressional Districts Intercept	0.020
State Intercept	0.111
Week Intercept	0.004

$^{***}p < 0.001$; $^{**}p < 0.01$; $^{*}p < 0.05$

Table S8. Multilevel logistic regression on Nationscape Survey Respondents from May 28 to Aug 11, 2020. For table readability, coefficient estimates of education, ethnicity and household income levels are not shown.
